# Solution Structure of the dATP-Inactivated Class I Ribonucleotide Reductase From *Leeuwenhoekiella blandensis* by SAXS and Cryo-Electron Microscopy

**DOI:** 10.3389/fmolb.2021.713608

**Published:** 2021-07-26

**Authors:** Mahmudul Hasan, Ipsita Banerjee, Inna Rozman Grinberg, Britt-Marie Sjöberg, Derek T. Logan

**Affiliations:** ^1^ Biochemistry and Structural Biology, Dept. of Chemistry, Lund University, Lund, Sweden; ^2^ Dept. of Biochemistry and Biophysics, Stockholm University, Stockholm, Sweden

**Keywords:** ribonucleotide reductase, allosteric regulation, oligomerization, nucleotide binding, small-angle X-ray scattering, single particle cryo-EM

## Abstract

The essential enzyme ribonucleotide reductase (RNR) is highly regulated both at the level of overall activity and substrate specificity. Studies of class I, aerobic RNRs have shown that overall activity is downregulated by the binding of dATP to a small domain known as the ATP-cone often found at the N-terminus of RNR subunits, causing oligomerization that prevents formation of a necessary α_2_β_2_ complex between the catalytic (α_2_) and radical generating (β_2_) subunits. In some relatively rare organisms with RNRs of the subclass NrdAi, the ATP-cone is found at the N-terminus of the β subunit rather than more commonly the α subunit. Binding of dATP to the ATP-cone in β results in formation of an unusual β_4_ tetramer. However, the structural basis for how the formation of the active complex is hindered by such oligomerization has not been studied. Here we analyse the low-resolution three-dimensional structures of the separate subunits of an RNR from subclass NrdAi, as well as the α_4_β_4_ octamer that forms in the presence of dATP. The results reveal a type of oligomer not previously seen for any class of RNR and suggest a mechanism for how binding of dATP to the ATP-cone switches off catalysis by sterically preventing formation of the asymmetrical α_2_β_2_ complex.

## Introduction

The enzyme ribonucleotide reductase (RNR) catalyzes the reduction of ribonucleotides to deoxyribonucleotides. Being the only source of dNTPs for DNA synthesis and repair, RNR is essential for all but a very few living organisms. Since its discovery in the 1950s, RNR has continuously delivered surprises in terms of radical chemistry, allosteric regulation and molecular organisation, which have all been extensively reviewed previously (Hofer et al., 2012; Ahmad and Dealwis, 2013; Torrents, 2014; Lundin et al., 2015; Mathews, 2018; Greene et al., 2020; Högbom et al., 2020). The very large family of RNR enzymes can be divided into three major functional classes based on their radical generation mechanisms (Högbom et al., 2020). All RNRs have a catalytic subunit with a 10-stranded α-β barrel fold, in class I called NrdA or α. Class I RNRs are activated by a radical generating subunit from the ferritin superfamily of all-α-helical proteins (NrdB or β). Class II RNRs, which can function both aerobically and anaerobically, have only a catalytic subunit and generate a 5′-deoxyadenosyl radical directly in the active site by homolytic cleavage of the C-Co bond in adenosylcobalamin. Class III, anaerobic RNRs also generate a 5′-deoxyadenosyl radical but do so by homolytic cleavage of S-adenosylmethionine by an accessory activase. This radical in turn generates a glycyl radical in the active site of the catalytic subunit. All three classes are unified by the transfer of these radicals to a cysteine next to the substrate in the active site.

**FIGURE 1:**
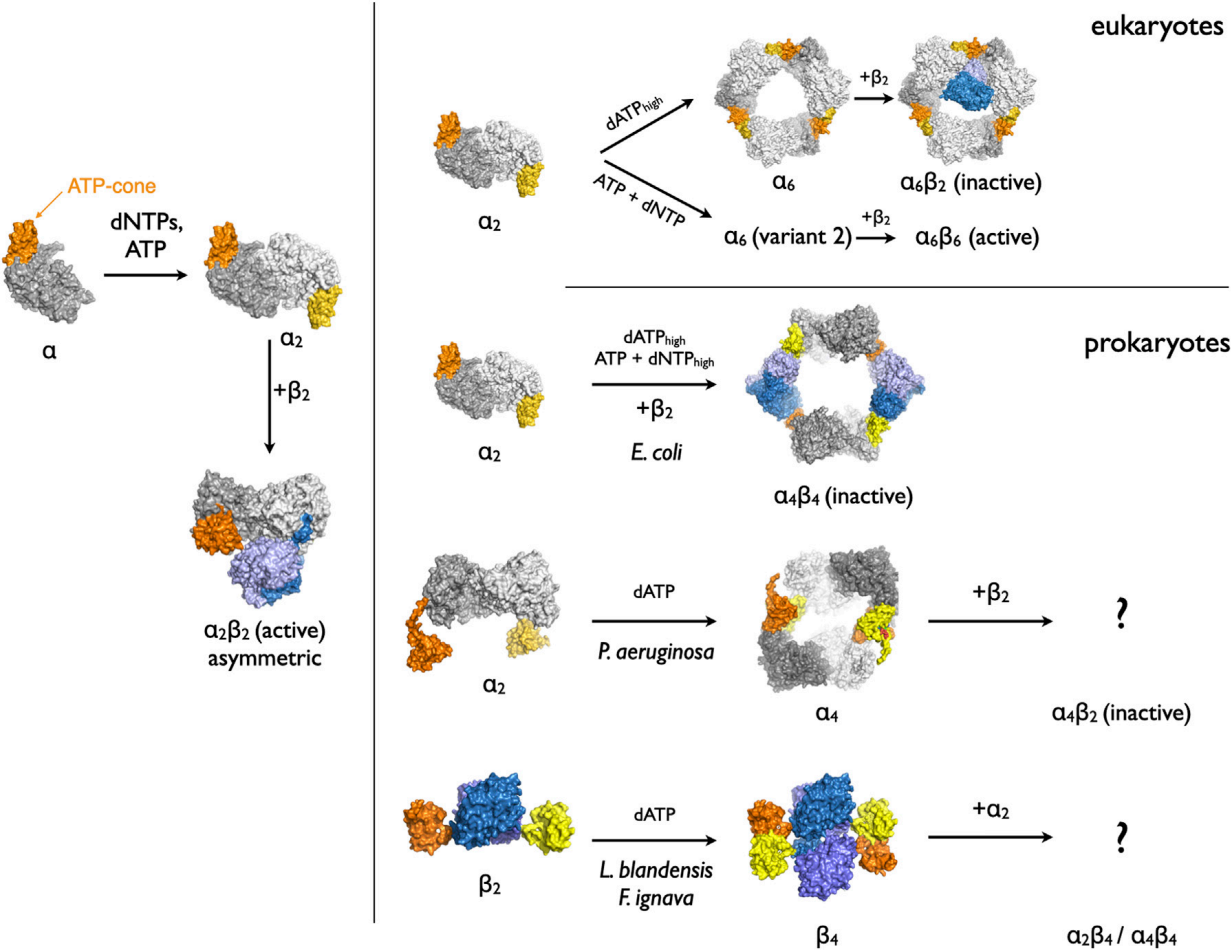
The variety of oligomers generated by binding of dATP to the ATP-cone in RNRs from different organisms. Low concentrations of dATP or other specificity-regulating dNTPs shift the monomer-dimer equilibrium towards dimers. Higher dATP concentrations induce the formation of inactive complexes of differing stoichiometries. The core domains of each NrdA subunit are shown in two shades of grey. The two ATP-cone domains in each dimer are shown in orange and yellow. The NrdB proteins are coloured in two shades of blue.

RNR proteins are regulated at two levels: overall activity and substrate specificity. RNRs are active in the presence of ATP and downregulated by dATP through binding of these nucleotides to a small, approximately 100-residue domain known as the ATP-cone (Aravind et al., 2000), most often found at the N-terminus of the catalytic α subunit. Over the past decade it has become evident that the ATP-cone, in the presence of dATP, directs the formation of a variety of catalytically incompetent oligomers (Figure 1), all of which have in common that they break the sensitive chain of residues necessary for the proton-coupled electron transfer (PCET) that must occur upon each cycle of catalysis. In *Escherichia coli,* two dimers of NrdA and two dimers of NrdB form a ring-shaped α_4_β_4_ octamer in which the β_2_ dimers are sandwiched between the α_2_ dimers in such a manner that the PCET chain is broken (Ando et al., 2011). In eukaryotic RNR, three dimers of NrdA form a ring-shaped α_6_ hexamer (Fairman et al., 2011; Brignole et al., 2018) to which one β_2_ dimer can attach (Rofougaran et al., 2006; Fairman et al., 2011; Ando et al., 2016), though the interactions with NrdB and how they break the PCET chain are not fully structurally characterized for this system. More recently, higher order oligomers such as helices have also been proposed both for class Ib RNRs with partial ATP cones (Thomas et al., 2019) and class Id RNRs lacking ATP cones (Rose et al., 2019).

Another type of ATP-cone was identified through bioinformatics, biochemical and structural studies of the class I RNR from *Pseudomonas aeruginosa* (PaNrdA), which belongs to the subfamily NrdAz having multiple ATP-cones (Johansson et al., 2016). Remarkably, in *P. aeruginosa* the ATP-cone domain was found to bind two molecules of dATP rather than the one molecule previously observed for the subfamily NrdAg (Jonna et al., 2015). Binding of dATP induces the formation of a ring-shaped α_4_ tetramer consisting of two PaNrdA dimers. Furthermore, the contacts between ATP-cone domains that lead to tetramer formation involve a different area of the surface of the ATP-cone to those in subfamily NrdAg.

In some organisms, the ATP-cone is not found at the N-terminus of the catalytic subunit NrdA but rather at the N-terminus of the radical generating subunit NrdB (Rozman Grinberg et al., 2018a). In these organisms the NrdA subunit lacks an ATP-cone. Biochemical and structural studies of the class I RNR from the marine bacterium *Leeuwenhoekiella blandensis* have shown that the ATP-cone responds to the binding of dATP by inducing formation of an unusual β_4_ tetramer of two NrdB dimers, held together only by the interaction of the ATP-cones (Rozman Grinberg et al., 2018a). The details of these interactions are remarkably similar to those seen in PaNrdA despite the fact that the ATP-cone is fused to a subunit completely different in size and fold. The ATP-cone thus appears to be a genetically transposable “molecular adhesive” that can induce protein-protein interactions somewhat independently of the subunit it is attached to. The *L. blandensis* NrdB (LbNrdB) was also found to belong to a family of NrdBs with a novel dimanganese metal center that does not use a tyrosyl radical. A high-valent Mn_2_(III,IV) radical is instead generated directly at the metal center, as shown both for LbNrdB the closely related *Facklamia ignava* NrdB (FiNrdB) (Rozman Grinberg et al., 2018a; Rozman Grinberg et al., 2019).

When an ATP-cone-containing β is co-incubated with α, inactive oligomers with stoichiometries α_2_β_4_ and α_4_β_4_ can be isolated using size exclusion chromatography in the presence of dATP, for both the *L. blandensis* system (Rozman Grinberg et al., 2018a) and the one from *F. ignava* (Rozman Grinberg et al., 2018b). These complexes are inactive, since activity assays demonstrated a K_i_ of 20 μM for dATP, and at ∼90 μM dATP the enzyme is fully inactive.

One important unresolved question is how the dATP-induced tetramerization of LbNrdB and NrdBs with similar ATP-cone fusions inhibits enzymatic activity, since in the dATP-induced LbNrdB tetramer, the face of NrdB that should interact with NrdA in the active α_2_β_2_ NrdAB complex is left exposed (Rozman Grinberg et al., 2018a). How then do the α_2_ dimers bind to the tetramer in such a way that the resulting complexes are inactive? In 1994, Uhlin and Eklund proposed a symmetrical docking model for the active α_2_β_2_ complex in *E. coli,* based on the assumption that the symmetry axes of the dimers would coincide and that there would be maximal alignment of residues in the long-range PCET pathway (Uhlin and Eklund, 1994). The LbNrdB tetramer would superficially seem to present no hindrance to formation of such an active complex. However, a recent structure of the active *E. coli* RNR holoenzyme (EcNrdAB) determined using cryo-electron microscopy showed that the active complex was in fact highly asymmetrical (Kang et al., 2020). One EcNrdA monomer makes extensive interactions with one EcNrdB monomer, in which large stretches of the C-termini of EcNrdA and EcNrdB become ordered in the interface, while the other pair of monomers make almost no interactions (Figure 1, lower left). One hypothesis as to how the tetramerization of LbNrdB via the ATP-cone inhibits enzymatic activity is by sterically blocking the formation of such an asymmetric complex. Thus one important question is the structural organisation of the inactive complexes of this class of RNRs and how they differ from those of active complexes.

Here we present a study of the low-resolution three-dimensional structures of the two components of *L. blandensis* RNR in the presence of the inhibitory nucleotide dATP, namely LbNrdA alone, LbNrdB alone, and the dATP-induced inactive RNR complex LbNrdAB with stoichiometry α_4_β_4_, using small-angle X-ray scattering and cryo-electron microscopy. The results reveal a quasi-symmetrical complex in which the dimer axes of the LbNrdA and LbNrdB dimers are almost aligned and are consistent with a model in which the ATP cone sterically prevents the formation of a more asymmetrical active complex.

## Materials and Methods

### LbNrdA Homology Model Preparation

A homology model for residues 40–596 of LbNrdA was made using the SwissModel server (Waterhouse et al., 2018) and the sequence of LbNrdA from UniProt (accession no. A3XHF8, gene MED217_17,130). The best template found was the 1.76 Å resolution crystal structure of the class Id NrdA from *Actinobacillus ureae* (PDB ID 6DQX) (Rose et al., 2019), which has 59% sequence identity to LbNrdA over 92% of the length of LbNrdA. In addition, models were generated using the Phyre2 (Kelley et al., 2015) and I-TASSER (Yang et al., 2015) servers. Geometry statistics for all models were evaluated using the SwissModel server, including analysis using MolProbity (Chen et al., 2010) and are presented in Supplementary Table 1, along with RMS deviations in Cα positions between the models.

### LbNrdA/LbNrdB Complex Model Preparation

The LbNrdA homology model obtained from SwissModel was placed on both sides of the LbNrdB tetramer complex (PDB ID 5OLK) in orientations compatible with the docking model for active class Ia RNR from *E. coli* proposed in 1994 (Uhlin and Eklund, 1994) by superimposing one of the dimers of 5OLK with the dimer of *E. coli* NrdB in the docking model, which was a gift from Ulla Uhlin and Hans Eklund.

### Expression and Purification of LbNrdA and LbNrdB

Both LbNrdA and LbNrdB were purified as described previously (Rozman Grinberg et al., 2018a), except that the 6-His tags on the N-termini of both proteins were not removed. Protein concentrations were determined by A_280_ measurements using a Nanodrop spectrophotometer (Thermo Scientific) using molar extinction coefficients of 91,135 and 46,870 M^−1^ cm^−1^ for LbNrdA and LbNrdB respectively, as calculated from ProtParam (Gasteiger et al., 2005). Further preparation of the protein samples for SAXS experiments is described below.

### SAXS Data Collection

SAXS experiments were carried out at beamline BM29 of the ESRF, Grenoble, France (Pernot et al., 2013) at a wavelength of 0.9919 Å. Data for LbNrdA with dATP were collected in batch mode. Dilution series of 5, 2.5, 1.25, and 0.625 mg/ml were prepared in buffer containing 50 mM Tris-HCl pH 7.6, 300 mM NaCl, 2 mM DTT, 5% glycerol, 2 mM magnesium acetate and 1 mM dATP, incubated for 5 min and centrifuged at high speed. The same buffer was used for buffer subtraction. Samples were loaded using the ESRF/EMBL SAXS sample changer (Round et al., 2015). Software implemented at the beamline was used for data collection, radial averaging of the images, background subtraction and conversion to 1D scattering profiles (Supplementary Figure 1). The protein solutions were flowed slowly through the quartz SAXS measurement capillary (diameter 1.5 mm) to avoid radiation damage and the temperature was kept constant at 10 °C. The scattered intensities were recorded on a pixel detector, Pilatus 1 M (Dectris), at a sample-detector distance of 2.867 m, allowing a range of momentum transfer q from 0.025 to 5.0 nm^−1^ (q = 4π sin Θ/λ, where 2q is the scattering angle and λ is the X-ray wavelength). Twenty frames of 45 ms each were recorded and compared to each other to check for radiation damage before merging. Further details are provided in Table 1.

**TABLE 1:** SAXS data collection and derived parameters. *Ab initio* models were calculated in expert mode where the number of knots used from the scattering curve was adjusted to make better agreement between the data and model. Structural parameters from crystal structures or homology model were calculated using CRYSOL.

Data collection parameters	LbNrdA + LbNrdB dATP	LbNrdA + dATP	LbNrdB + dATP
Experimental q range (nm^−1^)	0.0208–4.941	0.0208–4.941	0.0208–4.941
Exposure time (s)	0.045	0.045	0.045
Protein concentrations (mg ml^−1^)	n/a (SEC)	0.62, 1.25, 2.5, 5.0	n/a (SEC)
Structural parameters			
*I(0)* [from Guinier/*p(r)*]	298.86/302.20	79.51/79.51	176.06/175.6
*R*_g_ (nm) [from Guinier/*p(r)*]	6.36 ± 0.11/6.42 ± 0.11	3.84 ± 0.04/3.88 ± 0.02	4.51 ± 0.06/4.49 ± 0.03
Shell *R*_g_ (nm) from crystal structure or model	7.31	4.56	4.81
*D*_max_ (nm)	22.6 ± 0.2	13.37 ± 0.02	15.1 ± 0.1
Envelope diameter (nm) from crystal structure or model	19.4	12.2	13.2
Porod volume estimate (nm^3^)	724.06	221.93	375.67
Molecular mass determination			
Average excluded volume, *V*_ex_ (Å^3^)	726.159	210.832	378.044
Calculated monomeric *M*_r_ from sequence (kDa)	α: 70.66, β: 51.78 α_4_β_4:_ 489.76 α_2_β_4:_ 348.44	α: 70.66 α_2_: 141.32	β: 51.78, β_4_: 207.12
Mol. Mass from excluded volume (kDa)	427.15	124.02	222.38
Mol. Mass from porod volume (kDa)	425.91	130.55	220.98
Mol. Mass from SAXSMoW	466.5	141.7	254.4
Modelling parameters			
*Ab initio* Analysis	*GASBOR*	*GASBOR*	*DAMMIN*
Symmetry imposed	P222	P2	P222
Number of knots used	58	74	86
Validation and averaging	*DAMAVER*	*DAMAVER*	*DAMAVER*
χ^2^	1.38 ± 0.233	1.22 ± 0.125	0.99 ± 0.007
NSD value	1.035 ± 0.013	1.097 ± 0.035	0.726 ± 0.059
DENSS estimated resolution by FSC = 0.5 criterion (Å)	49.7	28.2	41.5

For the complex of LbNrdA and LbNrdB, as well as LbNrdB with dATP, in-line size exclusion chromatography with SAXS (SEC-SAXS) was employed, where purified protein sample was loaded onto a HiLoad 10/300 GL Superdex S200 (GE Healthcare) column. For the complex, NrdA and NrdB were mixed to reach a final concentration of 85 μM each, in buffer containing 50 mM Tris-HCl pH 7.6, 300 mM NaCl, 10% glycerol, 2 mM DTT, 1 mM dATP and 2 mM magnesium acetate. After 5 min incubation, the mixture was centrifuged at high speed and 350 μL were injected onto the column. The proteins were eluted at a flow rate of 0.5 ml min^−1^ and passed through the capillary cell. Frames were collected every 1 s with a total of 3,000 frames. The buffer for SEC-SAXS was 50 mM Tris-HCl pH 7.6, 300 mM NaCl, 2 mM DTT, 1 mM Mg acetate, 0.4 mM dATP and 5% glycerol. LbNrdB with dATP was prepared in the same buffer as the LbNrdA-LbNrdB complex, 350 μL of 11 mg/ml protein were loaded into the column and the same flow rate and SEC buffer were used in the SEC-SAXS data collection. Total scattering profiles and Rg plots for the elutions are shown in Supplementary Figures 2, 3.

### SAXS Data Processing

The datasets collected in batch mode at different protein concentrations were compared to look for concentration-dependent aggregation effects. Having found no such artefacts, the datasets were then processed and scaled using Primus (Konarev et al., 2003). The radii of gyration (R_g_) of the proteins in solution were determined from the lowest q values of the SAXS data, using the Guinier approximation. The value I(0)/c, the scattering intensity at zero angle normalized to the protein concentration of the sample, is proportional to the molecular mass M of the protein, estimated after calibration of the intensity using reference samples. The distance distribution function, representing the distribution of distances between any pair of volume elements within the particle, together with the structural parameters derived from *p(r)*, i.e. the maximum dimension of the particle (*D*_max_), and the radius of gyration, were evaluated using the indirect transform method as implemented in the program GNOM (Guinier, 1939; Petoukhov et al., 2012). SEC-SAXS profiles were processed by CHROMIXS (Panjkovich and Svergun, 2018) and best fractions were chosen according to their expected elution volumes based on their molecular weight. The figures of envelopes from SAXS data and their corresponding molecular models were made using the programs PyMOL (www.pymol.org), Situs (Wriggers, 2012) and Chimera (Pettersen et al., 2004).

### *Ab initio* SAXS Modelling

The model calculations were performed using GASBOR (Svergun et al., 2001) or DAMMIN (Franke and Svergun, 2009). The scattering profiles up to q_max_ = 1.26 to 4.2 nm^−1^ were used and 20 independent runs of modelling were carried out with each method. The 20 models were averaged and filtered using DAMAVER (Volkov and Svergun, 2003). As an additional check on the oligomeric state of LbNrdA and LbNrdB and their complexes by GASBOR or DAMMIN, several ab-initio dummy residue reconstructions were performed assuming monomeric as well as other oligomeric organizations. In all cases the models were generated assuming that either symmetry (P2 or P222) or no symmetry was present, and the best results were selected.

### Electron Density Map Generation Using DENSS

As an additional check of the correctness of the ab initio structures obtained using GASBOR and DAMMIN, electron density maps were generated using the phase retrieval method implemented in the program DENSS (Grant, 2018). The same output pair distribution functions and D_max_ values from GNOM as used for DAMMIN and GASBOR were used. Twenty independent models were generated for each dataset. When appropriate, 2-fold symmetry was applied to the simulation (P222 is not possible in DENSS). The best principal axis of the ellipsoid describing the molecular dimensions to which to apply the symmetry was determined by applying it to each axis in turn in independent simulations.

### Cryo-Electron Microscopy

#### Sample Preparation

For making the LbNrdAB complex, 230 μL of LbNrdB were taken from an initial stock solution of 6 mg/ml, 2 mM dATP was added followed by 170 μL of LbNrdA from an initial stock solution of 10 mg/ml. The mixture was incubated for 10 min on ice. This was followed by centrifugation of the sample at 5,000 rpm for 6 min. Protein was initially present in buffer containing 50 mM Tris (pH 7.6), 50 mM NaCl, 2% glycerol, 2 mM MgCl_2_, 2 mM TCEP and 0.4 mM dATP. A Superdex 200 column was used for the purification of the complex. Gentle glutaraldehyde crosslinking was used, as initial trials showed that the complex fell apart almost completely on cryoEM grids. The original buffer was changed from 50 mM Tris to 50 mM HEPES as the Tris would react with the glutaraldehyde. Buffer exchange was done on the complex using an Amicon Ultra 4 ml centrifugal filter membrane. All other buffer components remained the same. Following wash of the Superdex 200 column using HEPES buffer, 4 ml of 0.05% glutaraldehyde were injected into the column. A further 2 ml of HEPES buffer (without glutaraldehyde) were then injected, followed by injection of the protein complex and elution. Fractions of 200 μL were collected. The formation of α_4_β_4_ complexes (theoretical molecular weight 490 kDa) was verified by the presence of a band at > 460 kDa on a Coomassie-stained reducing SDS-PAGE gel (not shown).

Single particle cryoEM grids were prepared using Quantifoil R 1.2/1.3 grids (without continuous carbon coating) using sample concentration of 2 mg/ml. The Vitrobot settings were T = 4°C, humidity 100%, blot time 5 s, blot force -5, wait and drain time 1 and 0 s respectively. The grids had a good particle distribution in thin ice.

#### Data Collection

Data were collected from the grids using a Titan Krios microscope (ThermoFisher) equipped with a Gatan K2 detector. A total of 493 movies were collected in counting mode using an accelerating voltage of 300 kV. The data acquisition parameters were as follows: objective aperture 100, energy filter slit 20 eV, illuminated area 1.02 μm, spot size 6 nm, spherical aberration 2.7, defocus range −1.5 to −1.3 μm, pixel size 0.82 Å^2^, dose 11.77 e^−^Å^−2^s^−1^ with an exposure time of 4 s, making a total dose of 47.1 e^−^Å^−2^. The total number of dose fractions was 40.

#### Data Processing

Data processing was done using cryoSPARC v2.15 (Punjani et al., 2017; Punjani, 2020). A total of 493 movies were imported then patch motion correction (multi) and patch CTF estimation (multi) were carried out using default parameters. A volume of a hypothetical octamer was made with one pair of LbNrdA dimers from the homology model and one LbNrdB tetramer from the crystal structure, based on the docking model of active complex suggested by Uhlin and Eklund, and was imported into cryoSPARC. From this volume, a set of 50 equally spaced templates was created for template-based particle picking from the CTF-corrected micrographs. The particle diameter was 210 Å and the minimum separation distance was 0.5 particle diameters. This template, low-pass filtered at 20 Å, gave 200,948 particles from the 493 micrographs. Particles with an NCC score <0.300 and power threshold <961 and >4,971 were then discarded, leaving 149,794 particles, which were then extracted with a box size of 448 pixels. All particles were used for 2D classification with 80 classes and default parameters. Fifteen 2D classes were selected. The 2D classes showed the tetramer of LbNrdB and one dimer of LbNrdA distinctly while the other dimer of LbNrdA seemed less well ordered. These 2D classes had 35,473 particles, which were used for a second round of 2D classification into 50 classes. From here, 27 2D classes having 29,902 particles were selected and used in two separate runs to make a single ab-initio model and two ab-initio models. The particles from the single model were then refined against the two models using heterogeneous refinement. One of the resulting models (model A) had 19,630 particles and a gold standard FSC resolution of 9.9 Å while the other had 10,272 particles with FSC resolution 10.8 Å. Finally, the 19,630 particles were refined against model A using homogeneous refinement, which produced a refined model with FSC resolution 8.2 Å.

### Alignment of Crystal Structures and Homology Models to *Ab Initio* Envelopes From SAXS

The bead models from DAMMIN and GASBOR, which were obtained from independent 20 averaged and filtered models, were converted to volumes using SITUS. The crystal structures and homology models were aligned to the resulting envelopes using a combination of rigid body fitting in Chimera and manual adjustment. Molecular structures were aligned to the DENSS volumes using the script denss.align.py and some manual adjustment.

## Results

### LbNrdA (α_2_) Homology Model

The LbNrdA construct used consists of 600 amino acids. No crystal structure of LbNrdA has been determined to date. A reliable homology model for residues 40–596 was prepared using the SwissModel server with the dimeric crystal structure of *Actinobacillus ureae* (PDB ID 6DQX) (Rose et al., 2019) as template. This NrdA has 59% sequence identity to LbNrdA over 92% of the length of the latter. The QMEAN score for the homology model was −1.20. Two further models were constructed using the Phyre2 and I-TASSER servers. All three models were evaluated using the same criteria on the SwissModel server. Comparative statistics are shown in Supplementary Table 1. The Phyre2 and I-TASSER models had poorer overall quality but predicted a structure for the N-terminal 39 residues based on weak homology to three helices in the “connector domain” (Parker et al., 2018; Rose et al., 2019) that falls between the ATP-cone and the core in many class Ia RNRs. However, this three-helix bundle packed against the core of the protein such that its hydrophobic core enclosed three charged residues from the surface of the core: Glu69, Arg72, and Asp76. Furthermore, the predicted 3-helix bundle clearly protrudes from the cryoEM reconstruction (see below). No homology to known structures could be found for the first 39 residues of LbNrdA using a BLAST search (Altschul et al., 1990). Taken together, the evidence points towards the N-terminal 39 residues of LbNrdA being disordered.

As expected, the homology model strongly resembles an NrdA dimer lacking an ATP-cone. The SwissModel coordinates were used for further interpretation of the interactions between LbNrdA and LbNrdB. The crystal structure with PDB ID 5OLK was used for NrdB (Rozman Grinberg et al., 2018a).

### Small-Angle X-Ray Scattering

SAXS data were collected for three samples: LbNrdA in the presence of dATP, LbNrdB in the presence of dATP, and finally the inactive complex of LbNrdA and LbNrdB in the presence of dATP (LbNrdAB). To ensure sample homogeneity, the full-length LbNrdB and LbNrdAB preparations were recorded using inline size exclusion chromatography. Key structural parameters derived from the SAXS data are presented in Table 1.

### LbNrdA Forms a Dimer in the Presence of dATP

**FIGURE 2:**
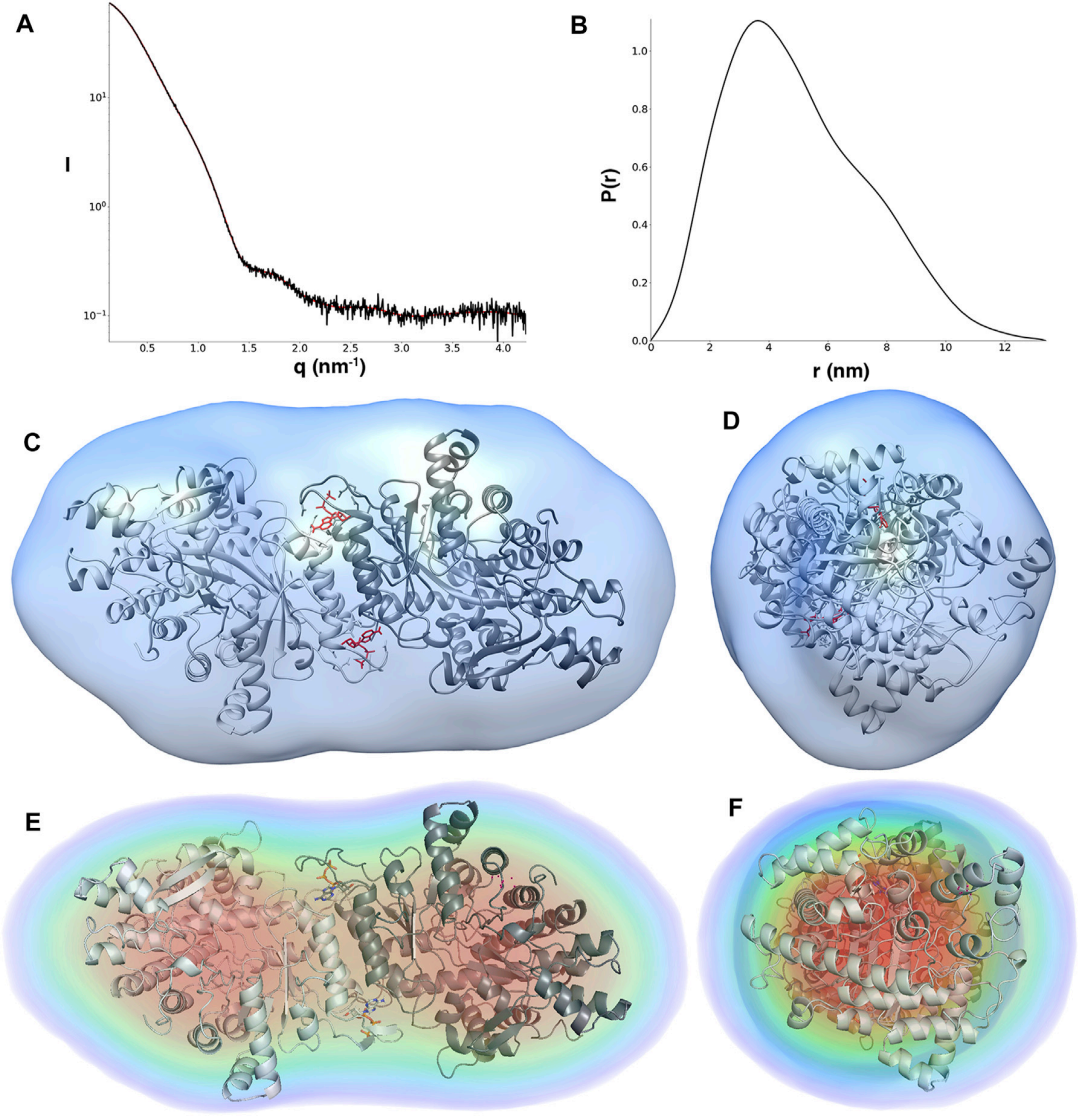
**(A)** Scattering curve (black line) and fitting (red line) to generate pair distance distribution function of dATP-bound LbNrdA. **(B)** Pair distance distribution function. The angular range used for fitting to generate *p*(r) is from 0.13 to 4.22 nm. *Ab initio* models of dATP bound LbNrdA superimposed on the homology model. **(C,D)** Two views of the envelope from GASBOR processed to a volume using SITUS, related by a 90° rotation around the vertical axis. **(E,F)** Similar views of the DENSS density reconstruction at approximately 42 Å resolution.

The solution scattering data from dATP-bound LbNrdA indicate a globular protein with an R_g_ of 3.92 nm and D_ma*x*_ of 13.9 nm (Table 1). The R_g_ value compares well with the value of 3.66 nm from the homology model, which lacks a total of 43 residues at the termini of each monomer, consistent with its lower R_g_. The distance distribution function is shown in Figure 2. The form of the *p(r)* function, with a maximum at less than *D*_max_/2, suggests that dATP-bound LbNrdA NrdA forms a species that is wider in one axis and the long tail on the right is consistent with the presence of unstructured terminal residues.

Furthermore, models for dATP-bound LbNrdA obtained by GASBOR *ab initio* reconstruction and DENSS phase recovery show a good fit to the dimeric homology model (Figure 2). The resolution of the DENSS reconstruction is estimated to be 28 Å by the FSC 0.5 criterion (Supplementary Figure 4). The correlation score of a 15 Å resolution map calculated from the homology model and the DENSS reconstruction is 0.889, as calculated in DENSS.

### LbNrdB in Complex With dATP Shows a Tetrameric Arrangement in Solution Consistent With the Crystal Structure

**FIGURE 3:**
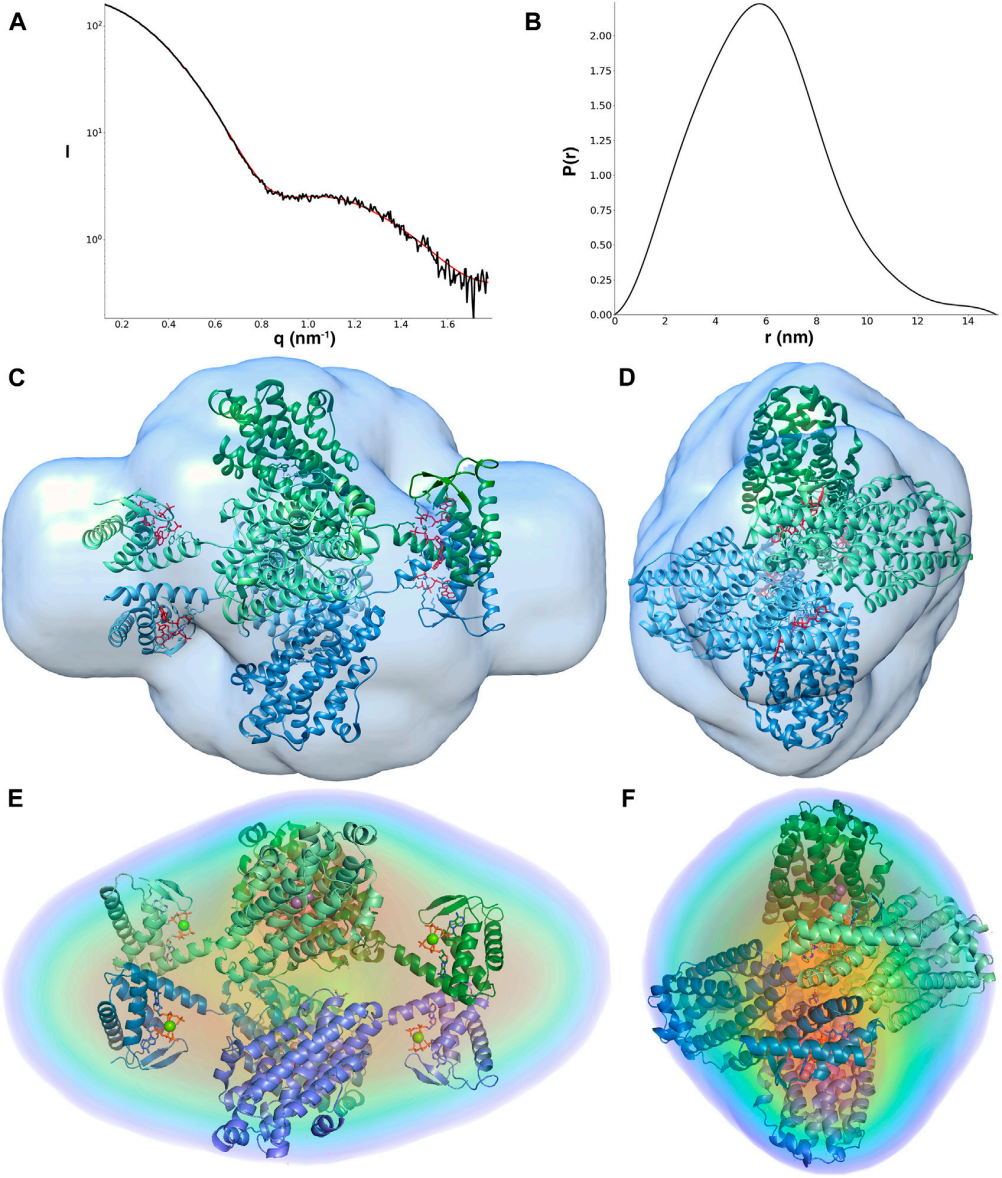
**(A)** Fit of the GASBOR model (red line) to the experimental scattering curve (black line); **(B)** pair distance distribution function of LbNrdB in the presence of dATP. The angular range used for fitting to generate *p*(r) was from 0.13 to 1.77 nm^−1^. **(C–F)** The tetrameric arrangement of LbNrdB as in PDB structure 5OLK fits well with the *ab initio* models derived from solution scattering data. **(C,D)** show the crystal structure fitted to the reconstruction from DAMMIN processed to a volume in SITUS. **(E,F)** show the fit of the crystal structure to the electron density reconstructed by DENSS at approximately 28 Å resolution. Views in **(D,F)** are rotated by 90° around the vertical axis relative to **(C,E)** respectively. The cartoon representation of LbNrdB is coloured by chain. The 8 dATP molecules are shown as sticks and the 4 Mg^2+^ ions as spheres.

SAXS data for LbNrdB in complex with dATP are shown in Figure 3. The *p*(r) function obtained from dATP-bound LbNrdB (Figure 3B) shows tailing at high values of r consistent with small separate domains connected to the ends of the core domain, i.e. the four ATP-cones of the tetramer. The distribution of *p*(r) peaks with a maximum at less than D_max_/2 also confirms the elongated form of dATP-bound LbNrdB. The R_g_ values of 4.49 nm obtained from the Guinier approximation and from the *p*(r) function are identical. These and the D_max_ value of 15.1 nm confirm the tetrameric arrangement in the known crystal structure (5OLK). The maximum distance between ordered atoms in the crystal structure is about 12.5 nm, thus D_max_ is consistent with the presence of disordered residues at the N- and C-termini (see below). The molecular mass calculated from the Porod volume is 221 kDa and the one calculated from the excluded volume is 222 kDa, both of which are close to the calculated one of 250 kDa, confirming the tetrameric arrangement of LbNrdB in solution.

The overall *ab initio* models obtained from the SAXS data also show good agreement with the crystal structure. The resolution of the DENSS reconstruction is estimated to be 42 Å (Supplementary Figure 4). The correlation score between the DENSS reconstruction and a 15 Å map calculated from the crystal structure is 0.740. The excluded volume obtained from the model is also consistent with a homotetrameric oligomeric state. The crystal structure of LbNrdB lacks between 24 and 29 residues at the N-terminus and 28–29 residues at the C-terminus of each chain, including the N-terminal His-tag and its cleavage site, which are not visible in the electron density due to flexibility. These regions may account for the extra volume close to the N-terminus (near the ATP-cone) and C-terminus.

### Modelling of the Missing N- and C-Terminal Residues in the LbNrdB Tetramer

The residues missing in the crystal structure at the N- and C-termini of each chain were modelled as dummy atoms using the program CORAL, which uses a fixed protein conformation with flexible ends and/or linkers. Thus, CORAL modelling gives good structural reconstructions for a more rigid and less flexible protein that does not possess a large structural heterogeneity. In contrast, CORAL yields poor reconstructions for systems that are better represented by a broader structural ensemble. In addition to the termini, the connecting neck between the N-terminal ATP-cone domain and C terminal domain consisting of 16 residues (from residue 103–118) was also deleted and rebuilt with CORAL.

**FIGURE 4:**
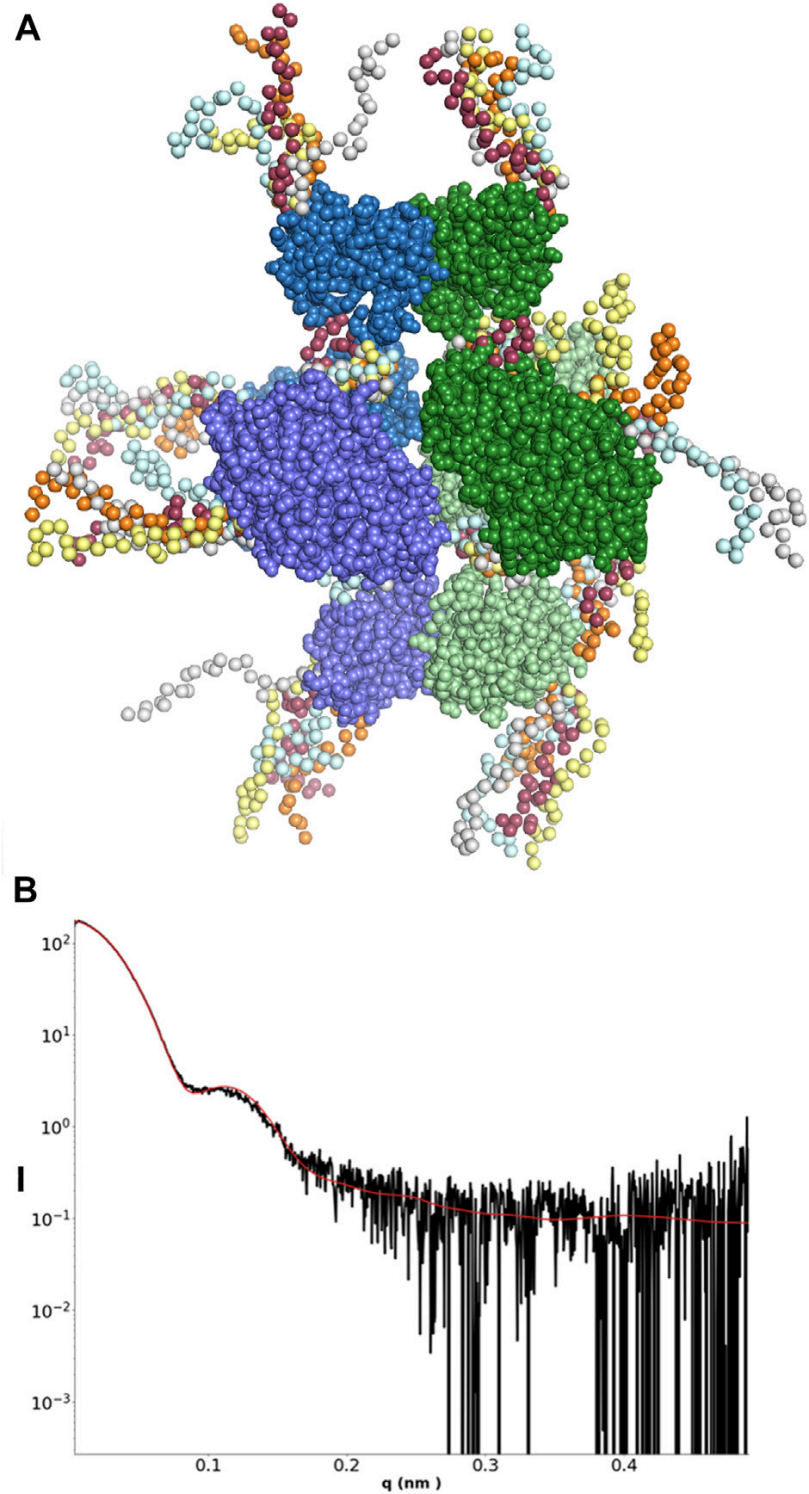
**(A)** Models of the LbNrdB tetramer with built missing N- and C-termini, each with 24–29 residues modelled using CORAL. The neck region between the core and ATP cone domains consisting of 16 residues was also deleted and rebuilt. Five possible models obtained from independent runs of CORAL are shown. **(C)** The scattering curve of LbNrdB (black) and its fit with the CORAL model (red).

CORAL was used to generate five representative models where χ^2^ values varied from 1.23 to 1.32 (Figure 4A). Through the CORAL modelling the χ^2^ value was reduced from on average 3.27 for the crystal structure alone to 1.27. This good fit (Figure 4B) indicates that the overall structure, including the relative orientations of the ATP-cone domains relative to the catalytic core domains, is very similar to the crystal structure and that the tetramer as a whole is not very flexible. In the CORAL model, in all four monomers the N-terminal residues project directly outwards from their respective ATP-cones. In contrast, the missing C-terminal residues show more variability in that they either lie close to the structured domains or project outwards in opposite directions. The modelled termini correspond well to the regions of density not occupied by the crystal structure in the DAMMIN and DENSS reconstructions.

### LbNrdA and LbNrdB Form a Hetero-Octamer With Stoichiometry α_4_β_4_ in Solution in the Presence of dATP

**FIGURE 5:**
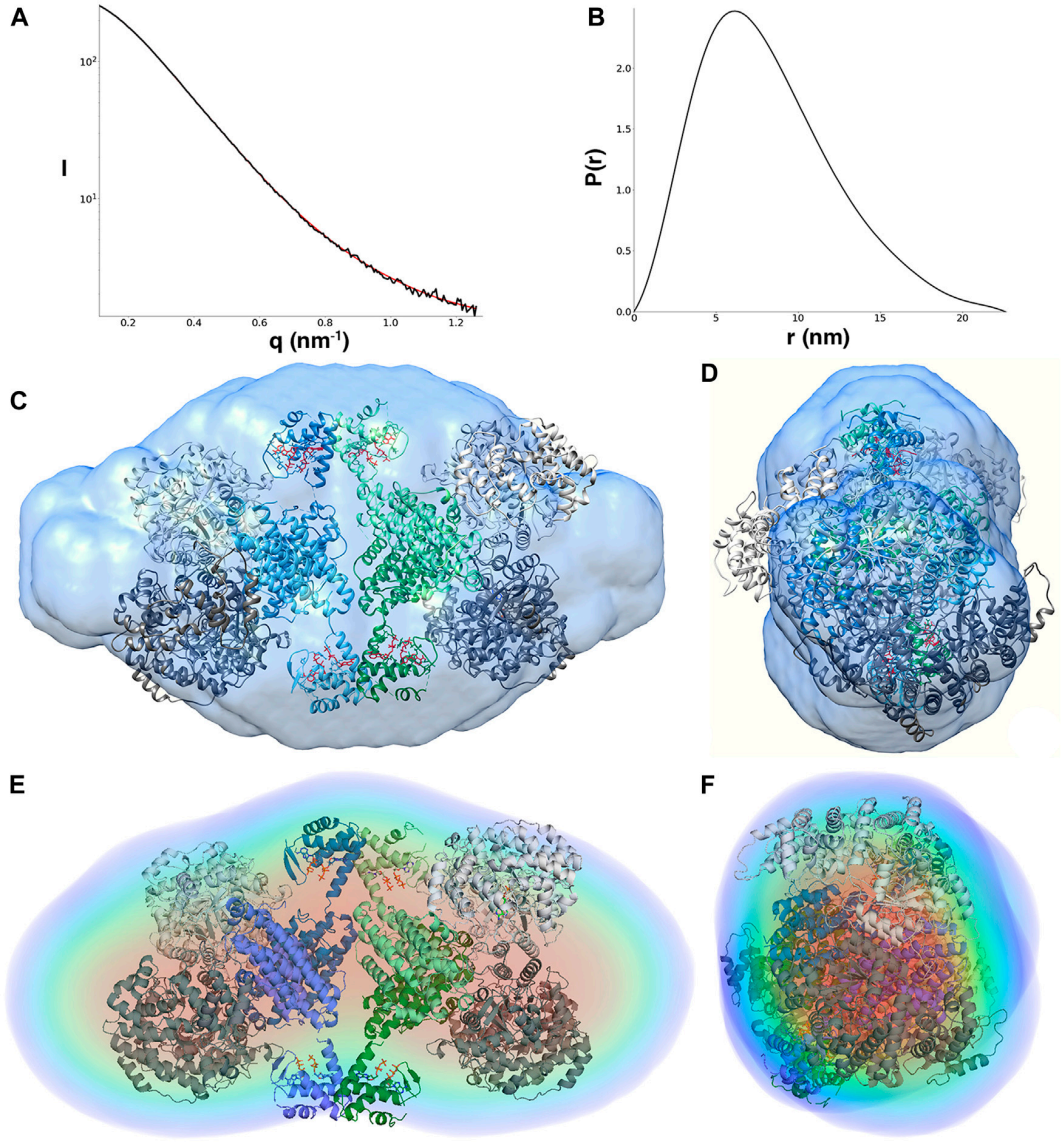
**(A)** Scattering curve of the LbNrdA/NrdB complex in the presence of dATP. **(B)** *p*(r) function. The angular range used to generate *p*(r) is from 0.11 to 1.26 nm. **(C,D)** Approximately orthogonal views of a fit of the docking model of the complex to the envelope from GASBOR. **(E,F)** Approximately orthogonal views of the fit of the same model to the DENNS reconstruction.

The Porod volume of the LbNrdAB species induced by dATP is 724.1 nm^3^, which indicates a molecular mass of 425.9 kDa. The calculated molecular mass of an α_4_β_4_ octamer that consists of the LbNrdB tetramer with two LbNrdA dimers attached is 489.8 kDa, while the molecular mass of an α_2_β_4_ hexamer is only 348.4 kDa. Thus the most plausible stoichiometry for this species in solution is α_4_β_4_, though we cannot exclude the presence of a small amount of α_2_β_4_. The R_g_ of the LbNrdAB complex is 6.26 nm and D_max_ is 22.6 nm, both of which are significantly larger than either of the β_4_ or α_2_ species described above. During ab-initio modelling with GASBOR, several possible symmetries were imposed and only P222 symmetry gave a reconstruction that fitted well with the data. This strongly supports an arrangement where two dimers of NrdA are arranged on opposite sides of the dATP-induced NrdB tetramer observed in the crystal structure and confirmed by SAXS (Figure 5). Furthermore, this arrangement is compatible with the concentration-dependent formation of α_4_β_4_ species observed in previous gas phase electrophoretic mobility analysis GEMMA and analytical SEC experiments (Rozman Grinberg et al., 2018a).

To test the compatibility of a symmetrical model for the inhibited α_4_β_4_ complex with the SAXS data, one copy of the homology model for the dimeric LbNrdA was placed on each side of the LbNrdB tetramer from the crystal structure in a way compatible with the docking model of the active complex of *E. coli* (Figure 5). This model fits reasonably well with the ab initio models, both from GASBOR and DENSS. The resolution of the DENSS model was estimated to be 50 Å (Supplementary Figure 4). The correlation score of a 15 Å map of the docking model with the DENSS volume was 0.768 as calculated by DENSS. Once again, the extra density present at both ends is most likely explained by the flexible N-terminus.

### CryoEM of the dATP-Inactivated LbNrdAB Complex

Analytical SEC had previously demonstrated the existence of species with molecular weights consistent with both α_4_β_4_ and α_2_β_4_ stoichiometries, with the stoichiometry being concentration-dependent (Rozman Grinberg et al., 2018a). Initial attempts were made to prepare cryoEM grids of the LbNrdAB complex by isolating fractions from a SEC run where LbNrdA had been mixed with excess LbNrdB. However, despite taking fractions from the centre of the peak in a well-defined elution profile that should correspond to the α_4_β_4_ complex, 2D classes generated from micrographs taken from these grids showed near-complete dissociation of the complex. We thus applied gentle glutaraldehyde cross-linking to the complex during the SEC run according to a published protocol previously applied to a GPCR/arrestin complex (Shukla et al., 2014). Here, the protein travels through a front of glutaraldehyde as it traverses the SEC column. Furthermore, the sample was applied to carbon-coated grids to maximize the amount of intact complex.

**FIGURE 6:**
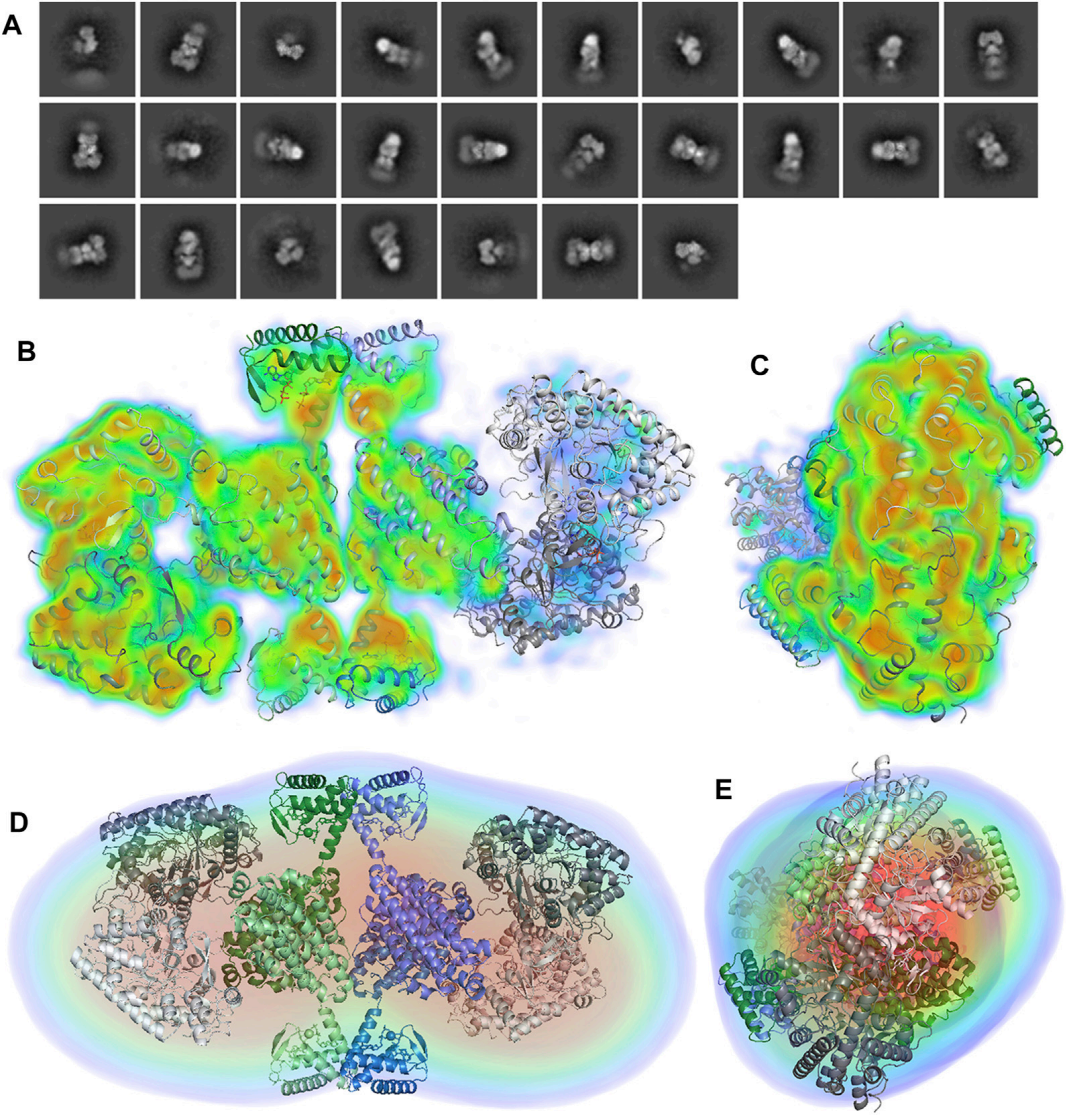
**(A)** Initial 2D classification of 29,902 particles of the cross-linked LbNrdAB complex in cryoSPARC. The first 27 classes are shown. **(B,C)** Approximately orthogonal views of the cryoEM volume of the α_4_β_4_ complex at 8.2 Å α_2_ dimers are shown in two shades of grey while the two dimers of the β_4_ tetramer are shown in in shades of green and blue respectively. The volume is coloured according to electrostatic potential level, from transparent blue at 2 σ through cyan at 6 σ, green at 8 σ, yellow at 10 σ to red at 15 σ, where σ is the number of standard deviations of the potential above the mean for the reconstruction box. Note the strong density for several helices. The lack of potential at higher levels for the right-hand dimer indicates lower occupancy and/or higher disorder. **(D,E)** Approximately orthogonal views of the fit of the cryoEM model to the SAXS envelope from DENSS (the GASBOR envelope is not shown for brevity).

Two-dimensional class averages from a small set of 29,902 particles (Figure 6A) suggest an organization similar to that revealed by SAXS, with one α_2_ dimer symmetrically bound to each side of a β_4_ tetramer. However, the sample is most likely a mixture of α_4_β_4_ and α_2_β_4_ species, as one of the α_2_ dimers is less well-ordered than the other, as already evident from the 2D classes (Figure 6A). The grids also contained a population of non-complexed β_4_ tetramers (not shown). A 3D reconstruction was made from these particles with a resolution of 8.2 Å according to a gold standard Fourier shell correlation value of 0.143 (Figure 6B and Supplementary Figure 5). No symmetry was imposed. The 3D volume confirms that one of the α_2_ dimers is significantly less well ordered or less occupied than the other, as the density is discontinuous and appears at a lower contour level than for the more ordered dimer (right hand side of Figure 6B). The dimeric homology model from the present work was fitted as a rigid body into the volume for α_2_ using Chimera and the crystal structure of the β_4_ tetramer was fitted into the corresponding volume as a single rigid body. Considering the more well-ordered of the two α_2_ dimers, it is arranged on top of one of the dimers of the β_4_ tetramer such that the active sites of both α_2_ monomers face the β subunits. However, the 2-fold axes of α_2_ and β_2_ are not exactly superimposed and the α_2_ dimer is tilted such that one of the monomers approaches one of the ATP-cones more closely. The shortest distance between two Cα atoms on one side is ∼10 Å, between Lys560 on α and Thr16 in β, while in the other pair of monomers the same atoms are ∼20 Å apart. Interestingly, neither of the ATP-cone domains apparently makes direct contact with the α_2_ dimers through hydrogen bonds or hydrophobic interactions. The molecular model fitted to the cryoEM map also fits at least as well as the docking model to the SAXS reconstruction from DENSS (Figures 6D,E).

**FIGURE 7:**
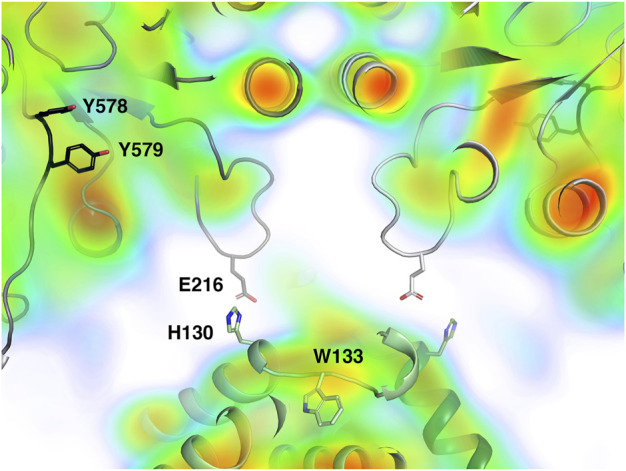
Closeup view of the central part of the interaction area between LbNrdA and LbNrdB in the dATP-inhibited complex, closest to the misaligned 2-fold symmetry axes. The homology model of LbNrdA and the crystal structure of LbNrdB are superposed on the 8.2 Å cryoEM reconstruction coloured as in Figure 6. Note the lack of electrostatic potential in the interface between the two proteins. The potential area of closest contact involves the loop containing Glu216 in the α subunit.

The distance between the last ordered amino acid in the PCET chain in α (the hydroxyl group of Tyr579; Figure 7) and the last ordered residue of the chain in β (the Cβ atom of Trp133) varies between 26 and 29 Å in the four pairs of interacting chains and there is no density in the 8.2 Å map that clearly suggests ordered structure at the interface between any α/β pair. In contrast, in the active complex from *E. coli*, the corresponding distance between the hydroxyl group of Tyr731 and the CB atom of Trp48 is only 19 Å and the intervening space is filled by the ordered C-terminus of the β subunit, including Tyr356, which is critical for the PCET pathway (Kang et al., 2020).

The closest approach between the α and β subunits occurs between the tip of the loop 213–222 between β-strands C and D of the α subunit and helix 118–131 on the β subunit (Figure 7). The distance between the Cα atom of Asp α216 and His β130 is around 10 Å. This interaction area is close to Trp133, one of the last residues in the PCET pathway on β. The side chains of α216 and β130 may be projected towards each other, but this interaction would be insufficient to stabilize the formation of a complex. It should be borne in mind that the α subunit in the current analysis is a homology model and that the interaction between subunits may involve structural elements not resolved at the current resolution. There is no well-defined density for this loop in the cryoEM reconstruction. Work is ongoing to deconvolute possible heterogeneity in the particle population and to obtain a larger dataset for higher resolution.

## Discussion

Class I ribonucleotide reductases are dependent for their activity on the formation of a productive complex between the α and β subunits to enable long-range proton-coupled electron transfer from β to α and back again on each catalytic cycle. The RNRs from *L. blandensis* and *F. ignava* are both inactivated by the formation of a tetramer of β subunits in the presence of dATP that is catalytically incompetent, most likely by holding the subunits in a relative orientation that breaks the PCET chain, as has been observed previously in various forms for other class I RNRs (Ando et al., 2011; Fairman et al., 2011). Here we have studied the oligomeric organization of the individual α and β components from *L. blandensis*, as well as the inactive complex, all in the presence of the inhibitory nucleotide dATP. The SAXS-derived structures agree well with a homology model for the α_2_ dimer and the previously published crystal structure of the β_4_ tetramer. Furthermore, the SAXS studies suggest that the ATP-cones of the β_4_ tetramer are not highly flexible in solution with respect to the core domains, and good agreement to the experimental scattering profile can be obtained simply by modelling the additional disordered residues at the N- and C-termini. Thus, the crystal structure is a faithful representation of the conformation in solution.

Biochemical and biophysical studies of the dATP-inactivated complex indicated the concentration-dependent formation of oligomers with stoichiometry α_2_β_4_ and α_4_β_4_ when α_2_ dimers were added to β_4_ tetramers (Rozman Grinberg et al., 2018a). However, the structures of these complexes, and thus the molecular mechanisms of inactivation of class II RNRs, in which the ATP-cone is found in the β subunit, have not been elucidated previously. Here we studied these complexes with both SEC-SAXS and cryoEM. The SAXS studies are consistent with a predominantly α_4_β_4_ stoichiometry and a hypothetical structure in which two α_2_ dimers attach symmetrically or pseudo-symmetrically to each side of a β_4_ tetramer. Such an arrangement would be inconsistent with an intact PCET pathway, as cryoEM studies on the class I RNR from *E. coli* have indicated that such a complex is in fact highly asymmetrical (Figure 1). However, the SAXS envelopes are of too low resolution to dissect the structure of the complex in detail.

To confirm and extend the solution results, we also studied the inactivated complexes by single particle cryoEM. The complexes were isolated using size exclusion chromatography in the presence of dATP using the same protocol as for SEC-SAXS. In addition, gentle on-column crosslinking using glutaraldehyde was applied. The latter was necessary because the forces exerted at the air-water interface in the cryoEM freezing entirely disrupted the complexes without crosslinking, though the SEC-SAXS results show that the same complexes are stable in solution. A reconstruction was obtained at 8.2 Å resolution that also reveals binding of two α_2_ dimers, one on either side of the β_4_ tetramer, with both active sites in the α_2_ dimer facing towards the active site of one β_2_ dimer, in a near-productive conformation. Despite cross-linking to prevent disruption of the complex during grid preparation, one of the dimers is less well-occupied than the other, but the SAXS results are most consistent with an α_4_β_4_, stoichiometry in solution. Most surprisingly, the cryoEM volume shows the subunits at some distance from each other and in a slightly asymmetrical orientation such that one ATP-cone on β_4_ approaches an α subunit more closely than does the other from the same dimer of the tetramer.

### Tetramerization May Inhibit *L. blandensis* RNR by Blocking Formation of an Asymmetric Active Complex

**FIGURE 8:**
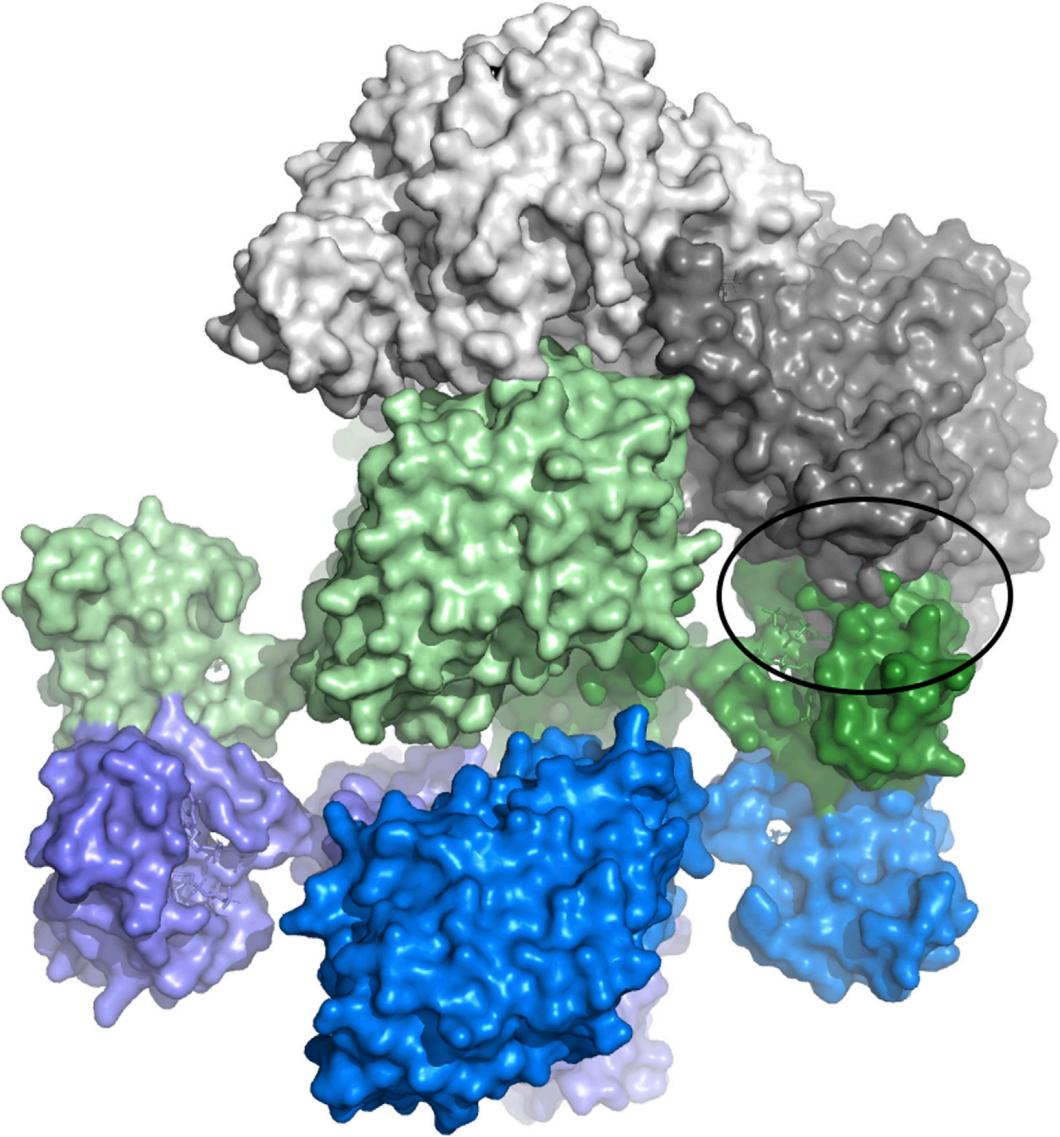
Model of the hypothetical steric clash that may prevent formation of an active complex of *L. blandensis* RNR when LbNrdB has tetramerized in the presence of dATP. Dimeric LbNrdA is shown in two shades of grey. The two dimers of LbNrdB that form the tetramer are shown in light and dark shades of green and blue respectively. The model has been generated to recreate the interactions that would occur in the active complex, based on the structure of *E. coli* NrdAB, between the light grey LbNrdA and pale green LbNrdB subunits. This orientation is prevented by a severe steric clash of the ATP-cone of the dark green LbNrdB subunit with the light grey LbNrdA subunit (circled).

The observation that the dATP-inhibited α_4_β_4_ LbNrdAB complex is quasi-symmetrical in combination with the recent discovery that the active complex of the *E. coli* enzyme is highly asymmetrical led us to hypothesize about how the inhibition might occur for *L. blandensis, F. ignava* and similar organisms. We used the homology model of LbNrdA in combination with the crystal structure of the dATP-inhibited LbNrdB tetramer and superimposed these on the cryoEM structure of the active *E. coli* α_2_β_2_ complex (EcNrdAB, PDB ID 6W4X (Kang et al., 2020)) to check for possible steric hindrances to the formation of an active complex (Figure 8). More specifically, we superposed the “active” monomer of the NrdB component of EcNrdAB (light green) on one chain of the LbNrdB tetramer. Then we superposed the homology model of LbNrdA (light grey) on the “active” monomer of EcNrdA from EcNrdAB. This provides a current best model of the interactions between LbNrdA and LbNrdB in the putative active complex. It can clearly be seen that in this conformation the ATP-cone of the “inactive” LbNrdB monomer (dark green in Figure 8) would clash with the “inactive” LbNrdA monomer (dark grey). Since the LbNrdA dimer is relatively rigid, this clash would also prevent the interaction of the two “active” chains.

Thus, despite subtle differences, our SAXS and cryoEM results are both compatible with the hypothesis that the ATP-cone functions as a steric block to formation of the highly asymmetrical complex that would be necessary for an intact PCET chain. These results begin to illuminate how the ATP-cone functions as a transposable functional module that, through oligomerization and steric hindrance, can inhibit RNR activity even when transposed from its usual place in the catalytic subunit to a new one in the radical generating subunit.

## Data Availability

The cryoEM data presented in this study can be found in the Electron Microscopy Database (EMDB) https://www.ebi.ac.uk/pdbe/emdb/, with accession number EMD-12985.
