# Supplementary material for: Solution Structure of the dATP-Inactivated Class I Ribonucleotide Reductase From Leeuwenhoekiella blandensis by SAXS and Cryo-Electron Microscopy
Source: Front Mol Biosci. 2021 Jul 26;8:713608. doi: 10.3389/fmolb.2021.713608 (PMC8350387; doi:10.3389/fmolb.2021.713608)
Supplement: Supplementary file 1 [file DataSheet1.docx]

Supplementary Material

# Supplementary Table

Table of geometric quality statistics for the three homology models generated of LbNrdA generated in this study.

|  | **Phyre2** | **SwissModel** | **I-TASSER** |
| --- | --- | --- | --- |
| **oligomeric state** | monomer | dimer | monomer |
| **residues modeled** | 1–600 | 40–596 | 1–600 |
| **MolProbity score** | 3.21 | 1.54 | 2.97 |
| **clash score** | 83.78 | 4.54 | 8.43 |
| **Ramachandran favored** | 91.8 | 95.6 | 83.6 |
| **Ramachandran outliers** | 2.3 | 0.6 | 5.5 |
| **Rotamer outliers** | 2.7 | 0.2 | 12.3 |
| **CB deviations** | 8 | 8 | 48 |
| **bad bonds** | 0 | 3 | 0 |
| **bad angles** | 125 | 115 | 91 |
| **cis-Pro** | 1 | 2 | – |
| **twisted non-proline** | 0 | 1 | 33 |
| **twisted proline** | 0 | 0 | 3 |
| **QMEAN** | -3.95 | -1.19 | -5.71 |
|  |  |  |  |
| **RMSD (Å) / atoms compared** | **Phyre2** | **SwissModel** | **I-TASSER** |
| **Phyre2** | – | 0.62 / 560 | 0.68 / 443 |
| **SwissModel** |  | – | 1.18 / 442 |
| **I-TASSER** |  |  | – |

# Supplementary Figures


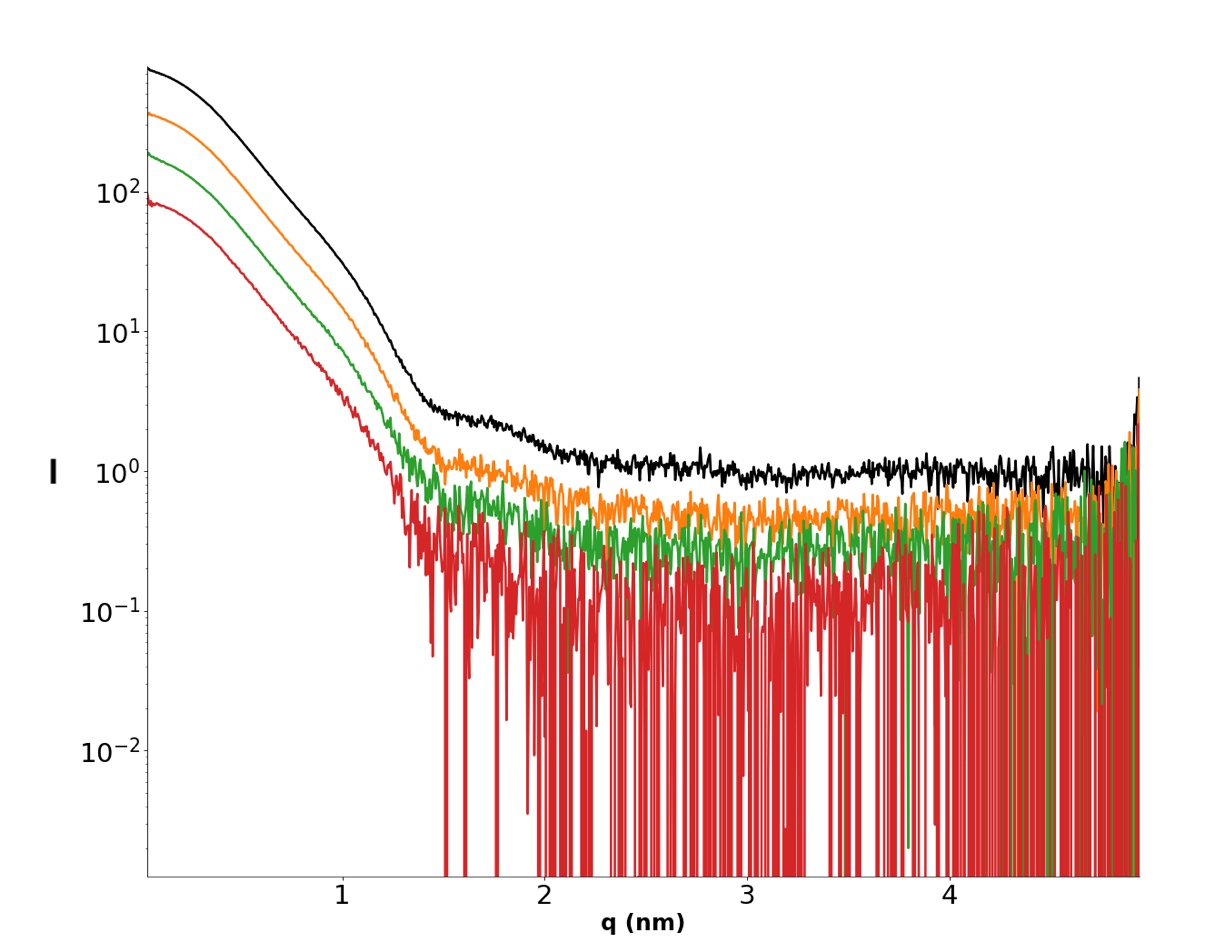


**Supplementary Figure 1:** Scattering profiles of dATP-bound LbNrdA at 5 mg/ml (black), 2.5 mg/ml (orange), 1.25 mg/ml (green) and 0.62 mg/ml (red).


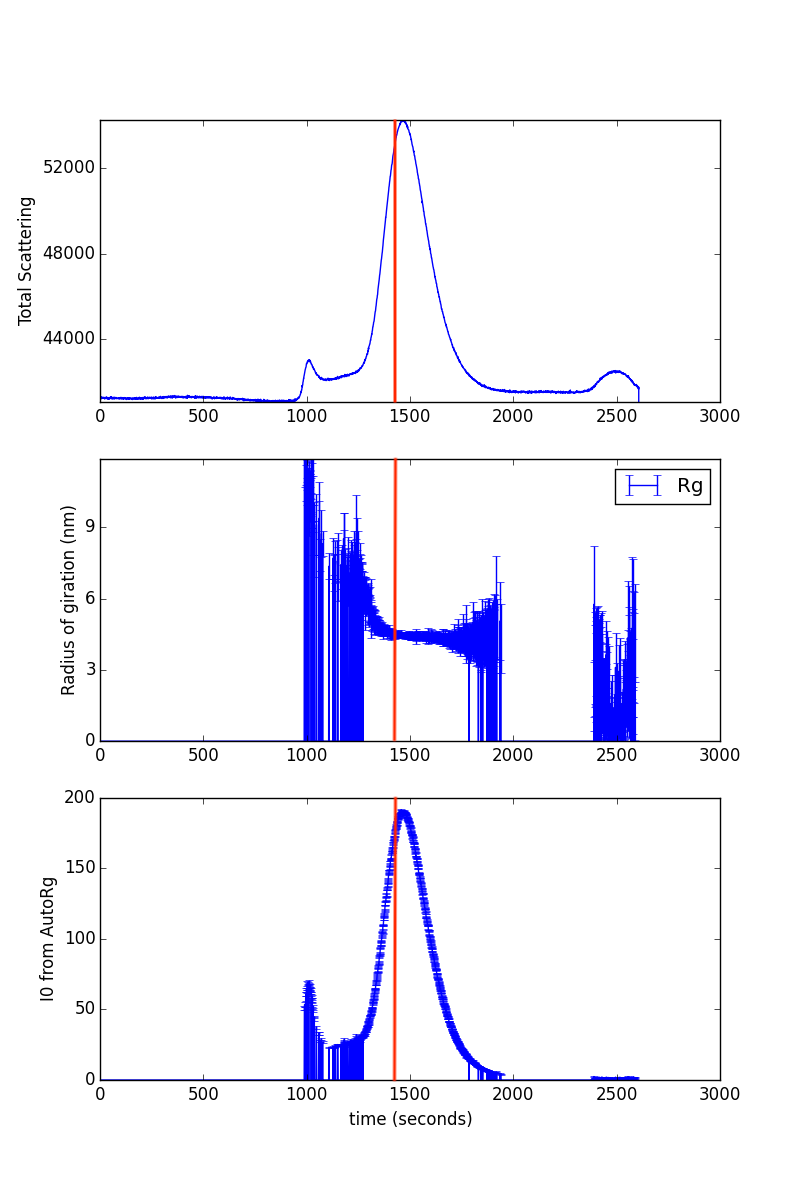


**Supplementary Figure 2:** a) The SEC-SAXS total scattering profile of LbNrdB with dATP. b) Automatic estimation of radius of gyration. Exposures 1416-1427 (marked by a red line) were used for the averaged profile.


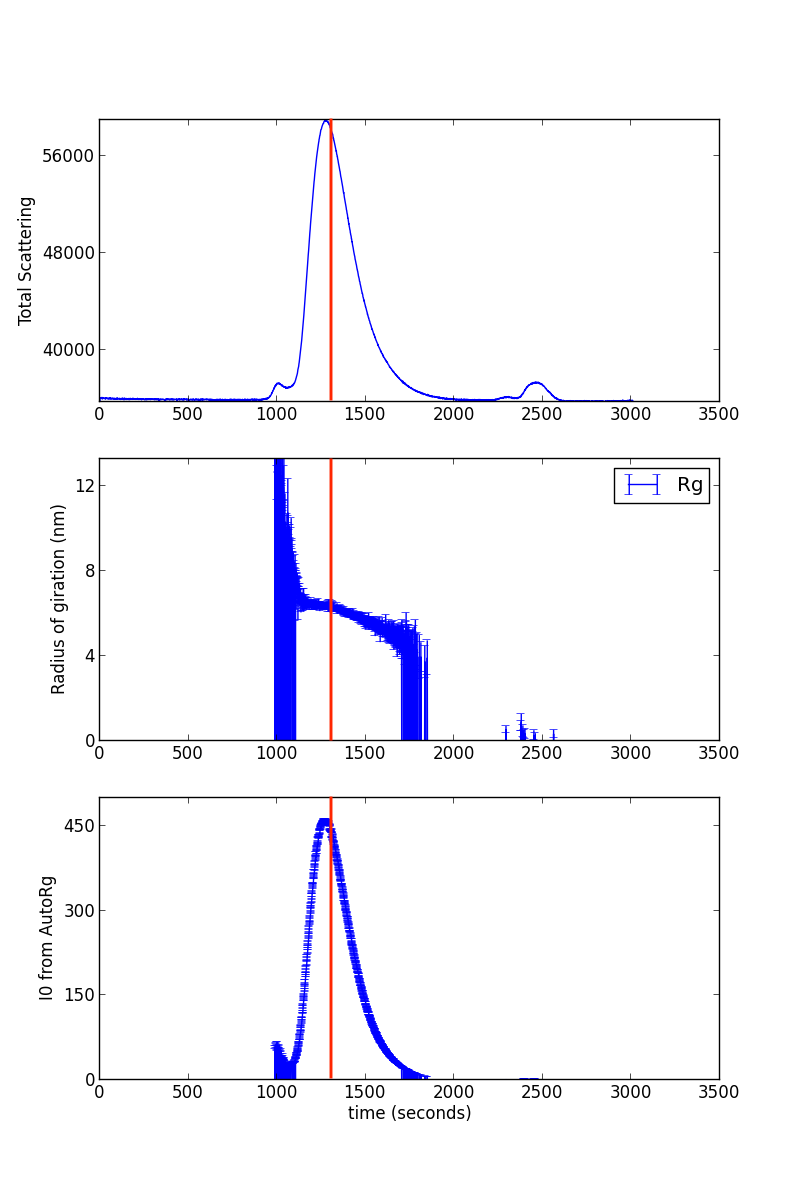


**Supplementary Figure 3**: a) The SEC-SAXS total scattering profile of the LbNrdAB complex with dATP. b) Automatic estimation of radius of gyration. Exposures 1302-1311 (marked with a red line) were used for the averaged profile.


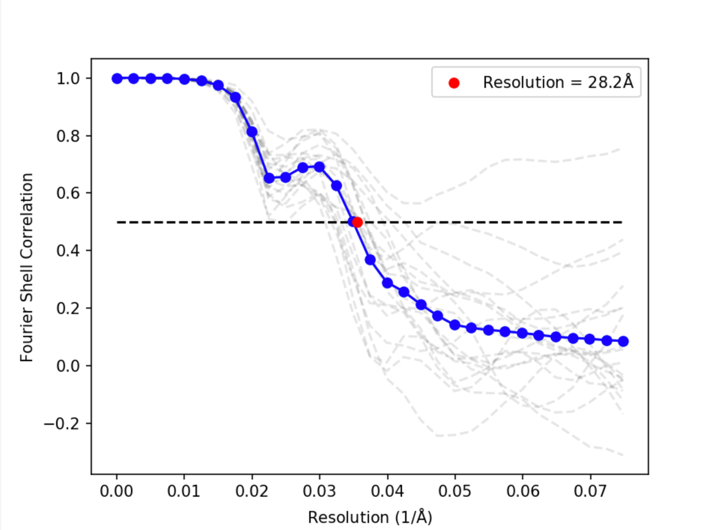

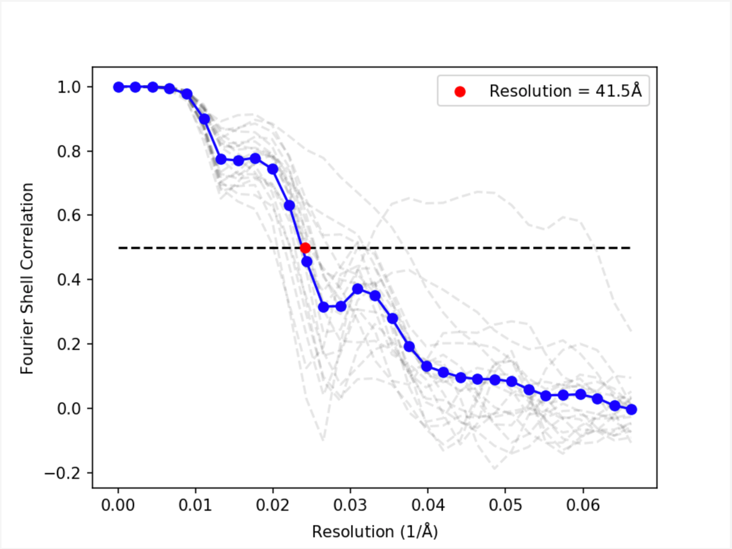

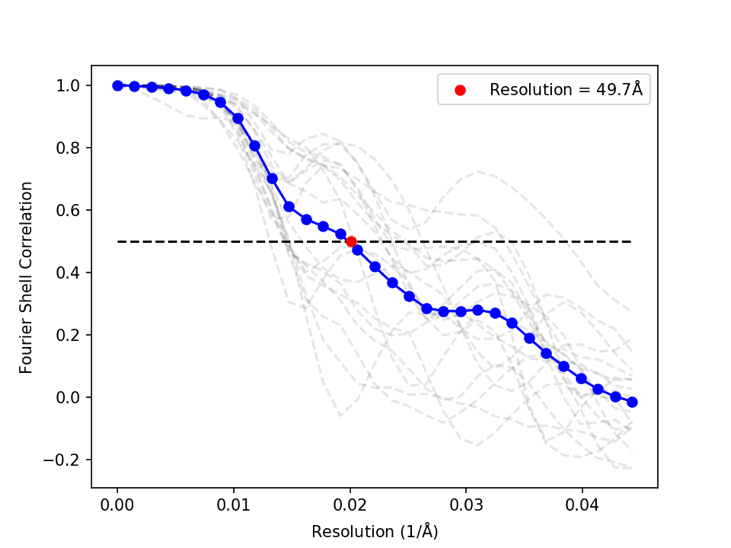


**Supplementary Figure 4:** Fourier shell correlation curves for the DENSS reconstructions. a) LbNrdA in the presence of dATP; b) LbNrdB in the presence of dATP; c) the LbNrdA/NrdB complex in the presence of dATP. The resolution limit indicated by the FSC = 0.5 criterion is shown as a red dot.


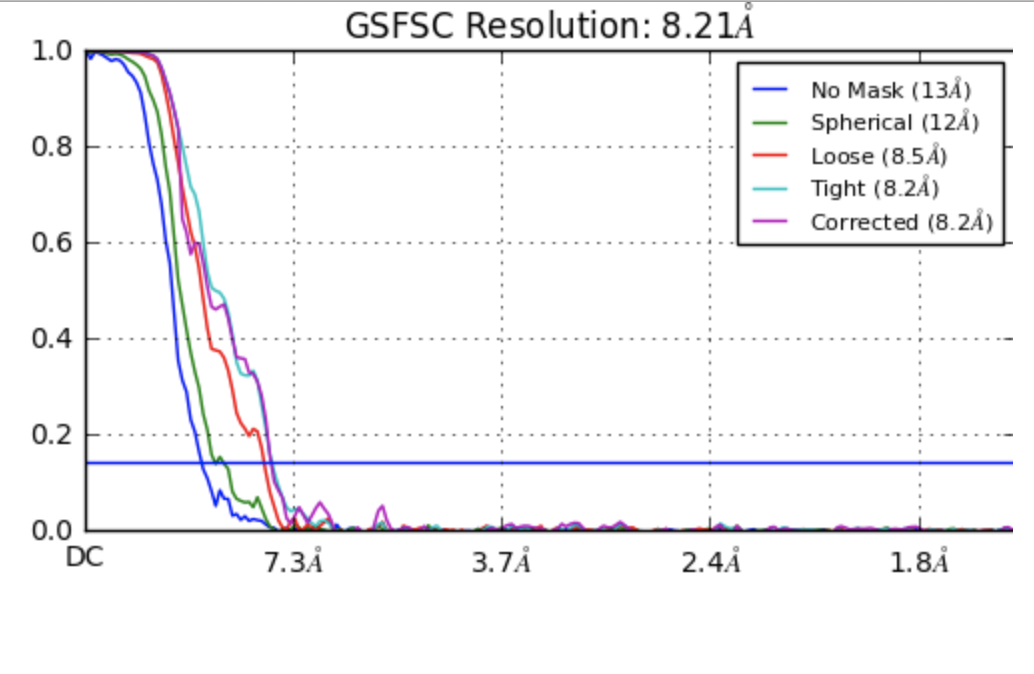


**Supplementary Figure 5:** Gold standard FSC curve (0.143 criterion) at 8.21 Å resolution from cryoSPARC for the cryoEM reconstruction shown in Fig. 6.
